# Supplementary material for: Bariatric Surgery Improves HDL Function Examined by ApoA1 Exchange Rate and Cholesterol Efflux Capacity in Patients with Obesity and Type 2 Diabetes
Source: Biomolecules. 2020 Apr 4;10(4):551. doi: 10.3390/biom10040551 (PMC7226587; doi:10.3390/biom10040551)
Supplement: Supplementary file 1 [file biomolecules-10-00551-s001.pdf]

Supplemental Figure S1

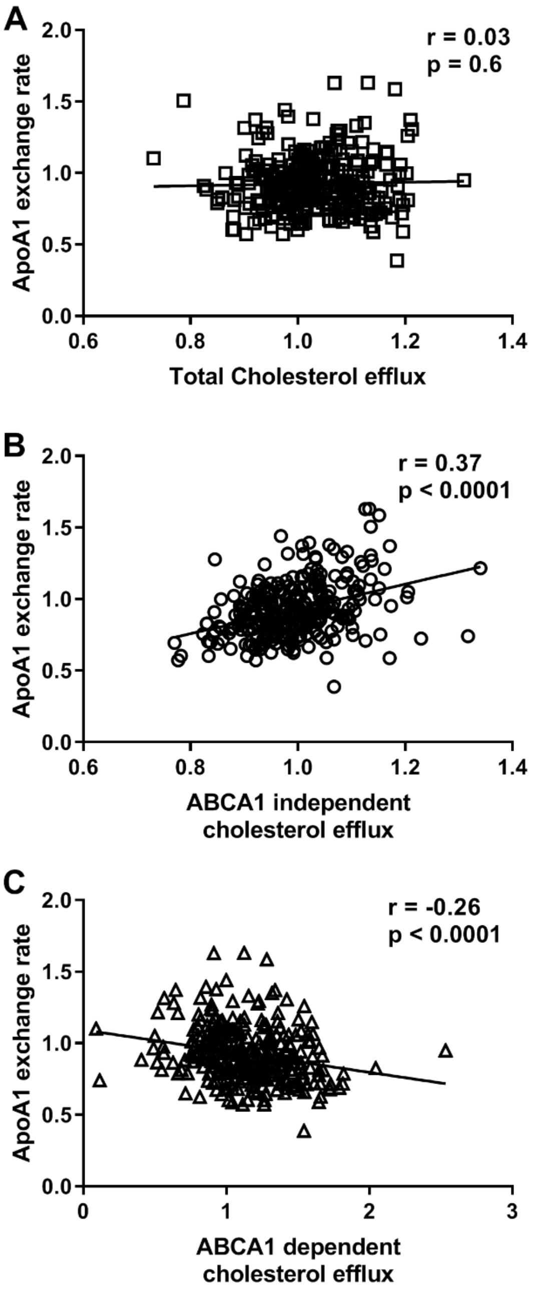

Supplemental Figure S1. Correlation of apoA1 exchange rate to cholesterol efflux capacity. ApoA1 exchange rate and cholesterol efflux capacity were assayed in 270 human serum samples from 90 Stampede patients. The total cholesterol efflux capacity which was measured when ABCA1 is induced (**A**), deducts ABCA1-independent efflux which was measured without ABCA1 induction (**C**), is the ABCA1 dependent cholesterol efflux capacity (**B**).

Supplemental Figure S2

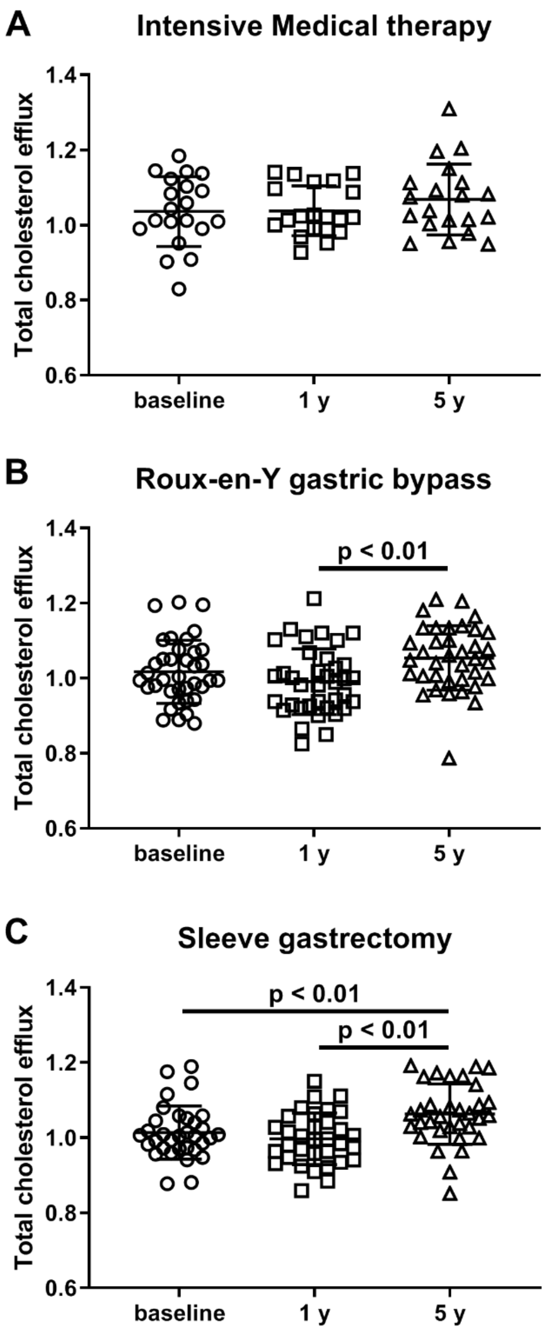

Supplemental Figure S2. Changes of total cholesterol efflux capacity after different treatments. The total cholesterol efflux capacity was measured in serum samples from patients who received intensive medical therapy (**A**, n=20), Roux-en-Y gastric bypass (**B**, n=37) or sleeve gastrectomy (**C**, n=33) surgeries at baseline, 1 year, and 5 years after the treatments.

Supplemental Figure S3

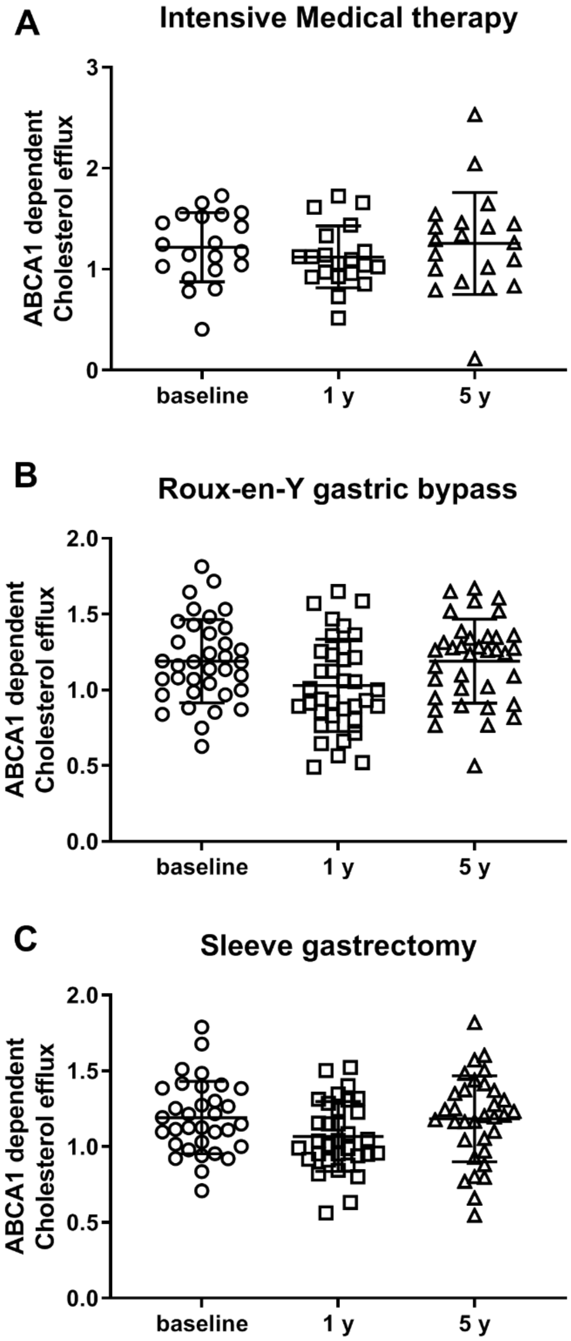

Supplemental Figure S3. Changes of ABCA1-dependent cholesterol efflux capacity after different treatments. ABCA1-dependent cholesterol efflux capacity was measured in serum samples from patients who received intensive medical therapy (**A**, n=20), Roux-en-Y gastric bypass (**B**, n=37) or sleeve gastrectomy (**C**, n=33) surgeries at baseline, 1 year, and 5 years after the treatments.

Supplemental Table 1. Multiple linear regression of changes of apoA1 exchange rate assay (AER) with changes in clinical variables at 1 year

Term	Coefficient	Std. Err.	T-statistic	p value
Intercept	-0.005	0.052	-0.098	0.92
Medical Therapy vs RYGB	-0.031	0.092	-0.337	0.74
Medical Therapy vs SB	-0.023	0.086	-0.262	0.79
HbA1c	-0.008	0.013	-0.609	0.54
BMI	-0.016	0.008	-1.897	0.061
log(TG)	0.027	0.044	0.622	0.54
**HDL**	**0.005**	**0.002**	**2.023**	**0.046**

Supplemental Table 2. Multiple linear regression of changes of ABCA1-independent CEC with changes in clinical variables at 1 year

Term	Coefficient	Std. Err.	T-statistic	p value
Intercept	0.033	0.02	1.629	0.11
Medical Therapy vs RYGB	-0.044	0.036	-1.219	0.23
Medical Therapy vs SB	-0.048	0.033	-1.441	0.15
HbA1c	-0.002	0.005	-0.476	0.64
BMI	-0.003	0.003	-1.05	0.3
**log(TG)**	**0.059**	**0.017**	**3.467**	**< 0.001**
**HDL**	**0.002**	**0.001**	**2.543**	**0.013**

Supplemental Table 3. Multiple linear regression of changes of apoA1 exchange rate assay (AER) with changes in clinical variables at 5 years

Term	Coefficient	Std. Err.	T-statistic	p value
Intercept	0.06	0.044	1.363	0.18
Medical Therapy vs RYGB	-0.079	0.058	-1.372	0.17
Medical Therapy vs SB	-0.025	0.058	-0.438	0.66
HbA1c	-0.009	0.009	-0.945	0.35
**BMI**	**-0.012**	**0.006**	**-2.086**	**0.041**
log(TG)	0.027	0.034	0.8	0.43
**HDL**	**0.004**	**0.002**	**2.809**	**0.006**

Supplemental Table 4. Multiple linear regression of changes of ABCA1-independent CEC with changes in clinical variables at 5 years

Term	Coefficient	Std. Err.	T-statistic	p value
**Intercept**	**0.054**	**0.027**	**2.046**	**0.045**
Medical Therapy vs RYGB	-0.059	0.035	-1.706	0.093
Medical Therapy vs SB	-0.055	0.035	-1.587	0.12
HbA1c	-0.007	0.005	-1.28	0.2
BMI	-0.004	0.003	-1.067	0.29
**log(TG)**	**0.049**	**0.021**	**2.352**	**0.022**
**HDL**	**0.003**	**0.001**	**3.398**	**0.001**
